# Supplementary material for: Attraction to similar options: The Gestalt law of proximity is related to the attraction effect
Source: PLoS One. 2020 Oct 28;15(10):e0240937. doi: 10.1371/journal.pone.0240937 (PMC7592845; doi:10.1371/journal.pone.0240937)
Supplement: S1 Table — (PDF) [file pone.0240937.s001.pdf]

S4 Table. Influence of the *value distance* on the choice proportion of the *target*.

	Experiment 1 (n = 38)				Replication (n = 81)			
**Fixed-effects Parameters**	***B***	**SE#**	***Z***	**p-val**	***B***	**SE#**	***Z***	**p-val**
Constant	0.19	0.05	3.74	<.001***	0.17	0.03	4.96	<.001***
Value distance	-0.25	0.14	-1.82	.07	-0.17	0.09	-1.80	.07
**Random-effects Parameters**	**var**				**var**			
Constant	0.00				0.00			
Value distance	0.16				0.17			

We used mixed effect logistic regression with random intercept and random slope.

Robust Std. Err. (Errors clustered by Subject); * p<.05 **p<.01 *** p<.001
