# Supplementary material for: Attraction to similar options: The Gestalt law of proximity is related to the attraction effect
Source: PLoS One. 2020 Oct 28;15(10):e0240937. doi: 10.1371/journal.pone.0240937 (PMC7592845; doi:10.1371/journal.pone.0240937)
Supplement: S2 Table — (PDF) [file pone.0240937.s002.pdf]

S5 Table. Summary of the mixed effects logistic regression model for variables predicting the choice proportion of the *target*.

	Experiment 1 (n = 38)				Replication (n = 81)			
**Fixed-effects Parameters**	***B***	**SE#**	***Z***	**p-val**	***B***	**SE#**	***Z***	**p-val**
Constant	0.52	0.13	3.95	<.001***	0.42	0.09	4.72	<.001***
Value distance	-0.25	0.14	-1.86	.06	-0.17	0.09	-1.82	.07
Gestalt Threshold	-0.04	0.02	-2.26	.02 *	-0.03	0.01	-2.72	<.01 **
Decoy Type (range/frequency)	-0.12	0.04	-2.97	<.01 **	-0.06	0.03	-2.11	<.05 *
**Random-effects Parameters**	**var**				**var**			
Constant	0.00				0.00			
Value distance	0.14				0.15			

Robust Std. Err. (Errors clustered by Subject); * p<.05 **p<.01 *** p<.001
