# Supplementary material for: Attraction to similar options: The Gestalt law of proximity is related to the attraction effect
Source: PLoS One. 2020 Oct 28;15(10):e0240937. doi: 10.1371/journal.pone.0240937 (PMC7592845; doi:10.1371/journal.pone.0240937)
Supplement: S1 Appendix — (PDF) [file pone.0240937.s003.pdf]

S1 Appendix. List of trials for the calibration task.

	Set 1					Set 2			
#	probA	amountA	probB	amountB		probA	amountA	probB	amountB
1	62	22	52	44		47	25	34	59
2	60	20	47	40		43	26	31	60
3	60	21	43	42		43	27	28	60
4	61	22	39	40		45	27	25	58
5	59	22	35	44		47	27	23	60
6	59	21	32	43		46	29	21	59
7	63	23	29	42		43	27	19	61
8	63	23	26	42		44	29	17	61
9	61	21	23	40		45	28	15	58
10	59	20	21	43		47	29	14	60
11	60	21	19	40		43	26	12	59
12	60	21	52	40		47	28	34	59
13	63	22	47	42		46	26	31	61
14	59	22	43	40		44	28	28	58
15	59	20	39	44		43	28	25	58
16	59	21	35	44		45	25	23	57
17	62	24	32	43		45	26	21	61
18	62	20	29	40		43	26	19	60
19	62	24	26	43		45	28	17	59
20	61	23	23	42		44	29	15	60
21	61	22	21	44		44	26	14	59
22	60	22	19	43		45	28	12	60
23	62	21	52	44		44	28	34	59
24	59	22	47	42		44	25	31	60
25	62	24	43	42		46	28	28	59
26	59	22	39	44		44	26	25	61
27	60	22	35	40		47	29	23	58
28	62	21	32	40		47	25	21	57
29	62	22	29	40		46	27	19	57
30	59	23	26	41		44	27	17	57
31	63	23	23	44		45	27	15	59
32	62	21	21	44		43	28	14	59
33	61	21	19	40		47	26	12	58
34	61	24	52	41		47	28	34	60
35	61	20	47	42		47	27	31	60
36	60	24	43	42		44	25	28	60
37	61	24	39	43		45	25	25	61
38	61	23	35	43		43	28	23	61
39	63	20	32	41		45	27	21	57
40	62	21	29	42		44	25	19	57
41	62	21	26	40		43	26	17	60
42	60	23	23	44		43	28	15	57
43	63	20	21	40		45	25	14	59
44	61	23	19	40		43	28	12	59
45	60	20	52	41		45	26	34	61
46	63	23	47	40		45	29	31	59
47	63	22	43	42		46	26	28	58
48	61	23	39	41		46	28	25	60
49	62	23	35	44		46	25	23	60
50	61	24	32	44		43	26	21	59
51	60	24	29	40		43	25	19	58
52	60	21	26	43		44	27	17	57
53	61	23	23	41		45	28	15	59
54	60	20	21	42		46	27	14	58
55	63	20	19	42		45	27	12	57
56	59	23	52	44		47	28	34	60
57	60	22	47	42		46	28	31	58
58	59	22	43	44		47	28	28	59
59	60	24	39	41		45	28	25	60
60	61	23	35	43		44	29	23	58
61	60	23	32	43		43	26	21	60
62	63	24	29	42		46	28	19	58
63	61	24	26	43		46	26	17	60
64	59	22	23	43		45	25	15	60
65	63	20	21	40		43	28	14	59
66	63	21	19	40		44	27	12	57
