# Supplementary material for: Attraction to similar options: The Gestalt law of proximity is related to the attraction effect
Source: PLoS One. 2020 Oct 28;15(10):e0240937. doi: 10.1371/journal.pone.0240937 (PMC7592845; doi:10.1371/journal.pone.0240937)
Supplement: S2 Appendix — (PDF) [file pone.0240937.s004.pdf]

S2 Appendix. Calculation of the decoy options.

Attribute (probability/amount) of the ***Decoy*** = Attribute of the ***Target*** **–** **[**Attribute of the ***Target*** **x Step(15%/30%/45%/60%)]**

Set 1: An example of decoys on the amount attribute for the 4 different steps of *value distance*

	Competitor	Target	Decoy(15)	Decoy(30)	Decoy(45)	Decoy(60)
Probability	45%	21%	21%	21%	21%	21%
Amount	27₪	59₪	50₪	41₪	32₪	24₪
EV	12.2	12.3	10.5	8.7	6.7	5

Set 2: An example of decoys on the probability attribute for the 4 different steps of *value distance*

	Competitor	Target	Decoy(15)	Decoy(30)	Decoy(45)	Decoy(60)
Probability	61%	32%	27%	22%	18%	13%
Amount	22₪	42₪	42₪	42₪	42₪	42₪
EV	13.4	13.4	11.4	9.4	7.3	5.3
