# Supplementary material for: Attraction to similar options: The Gestalt law of proximity is related to the attraction effect
Source: PLoS One. 2020 Oct 28;15(10):e0240937. doi: 10.1371/journal.pone.0240937 (PMC7592845; doi:10.1371/journal.pone.0240937)
Supplement: S3 Appendix — (PDF) [file pone.0240937.s005.pdf]

S6 Appendix. Individual results of binomial tests for choice proportion of *target*.

Experiment 1

#	Subject	Choice proportion of *target*	P-value
1	1	0.532	0.345
2	2	0.553	0.103
3	4	0.500	1.000
4	5	0.514	0.707
5	7	0.512	0.754
6	8	0.539	0.235
7	11	0.610	0.001
8	12	0.535	0.288
9	13	0.510	0.802
10	14	0.514	0.707
11	15	0.498	1.000
12	16	0.471	0.381
13	17	0.459	0.210
14	18	0.551	0.117
15	19	0.565	0.044
16	20	0.524	0.487
17	21	0.538	0.254
18	22	0.617	0.000
19	24	0.413	0.007
20	25	0.550	0.157
21	26	0.677	0.000
22	27	0.547	0.150
23	28	0.537	0.260
24	29	0.504	0.950
25	30	0.522	0.525
26	31	0.578	0.015
27	34	0.375	0.000
28	35	0.468	0.343
29	36	0.537	0.260
30	38	0.480	0.574
31	39	0.480	0.572
32	40	0.538	0.258
33	41	0.542	0.209
34	43	0.512	0.753
35	44	0.514	0.703
36	50	0.486	0.707
37	51	0.530	0.379
38	52	0.561	0.060

Replication

#	Subject	Choice proportion of *target*	P-value
1	2	0.430	0.032
2	3	0.498	1.000
3	4	0.498	1.000
4	6	0.598	0.002
5	8	0.450	0.130
6	9	0.532	0.345
7	10	0.502	1.000
8	11	0.553	0.103
9	12	0.567	0.037
10	14	0.500	1.000
11	15	0.522	0.531
12	16	0.549	0.131
13	17	0.508	0.851
14	18	0.555	0.091
15	19	0.508	0.850
16	20	0.590	0.005
17	21	0.520	0.574
18	22	0.529	0.381
19	23	0.831	0.000
20	24	0.500	1.000
21	25	0.482	0.616
22	26	0.514	0.707
23	27	0.502	1.000
24	29	0.531	0.349
25	30	0.508	0.851
26	32	0.469	0.347
27	33	0.482	0.616
28	35	0.606	0.001
29	36	0.537	0.260
30	37	0.506	0.900
31	39	0.500	1.000
32	40	0.604	0.001
33	41	0.584	0.008
34	42	0.477	0.492
35	43	0.512	0.754
36	44	0.523	0.492
37	45	0.539	0.235
38	46	0.502	1.000
39	48	0.514	0.707
40	49	0.473	0.443

41	50	0.506	0.900
42	52	0.527	0.417
43	53	0.610	0.001
44	54	0.576	0.017
45	55	0.506	0.900
46	56	0.506	0.900
47	57	0.535	0.288
48	58	0.510	0.802
49	59	0.539	0.235
50	60	0.514	0.706
51	62	0.525	0.452
52	63	0.492	0.851
53	65	0.584	0.009
54	67	0.471	0.381
55	68	0.464	0.314
56	69	0.558	0.077
57	70	0.461	0.235
58	71	0.561	0.060
59	72	0.459	0.210
60	73	0.530	0.379
61	74	0.551	0.117
62	75	0.565	0.044
63	77	0.524	0.487
64	78	0.413	0.007
65	79	0.538	0.254
66	80	0.550	0.157
67	81	0.617	0.000
68	82	0.486	0.707
69	85	0.514	0.703
70	87	0.512	0.753
71	91	0.677	0.000
72	92	0.542	0.209
73	93	0.547	0.150
74	94	0.538	0.258
75	95	0.537	0.260
76	96	0.480	0.572
77	97	0.504	0.950
78	98	0.468	0.343
79	99	0.522	0.525
80	100	0.422	0.015
81	101	0.578	0.015
