# Supplementary material for: Attraction to similar options: The Gestalt law of proximity is related to the attraction effect
Source: PLoS One. 2020 Oct 28;15(10):e0240937. doi: 10.1371/journal.pone.0240937 (PMC7592845; doi:10.1371/journal.pone.0240937)
Supplement: S1 Text — (PDF) [file pone.0240937.s006.pdf]

S3 Text. Additional analyses

1. Robustness analyses

We used mixed-effect logistic regressions in our main analyses, which requires a binary dependent variable per trial. This is the reason we chose to use the choice of either *target* or *competitor* as a measurement per trial for each subject and the choice proportion of the *target* overall trials (excluding trials in which the *decoy* option was chosen – 1% of the trials) as a measurement per subject.

In order to examine if our measurement (choice proportion of the *target)* is consistent with other measurements that are used in the literature, we measured the *attraction effect* with additional three measurements: violation of WASRP (the Weak Axiom of Stochastic Revealed Preference) [1, 2], violation of regularity [3] and *relative choice share of the target* (RST) [4, 5]. We chose specifically these measurements since: 1) the two first measurements refer to the two violations that are induced by the *attraction effect* [1, 2] and, 2) all of these measurements were previously used to measure the strength of the *attraction effect* [2, 4-7]. It is important to note that we did not pre-register these analyses. Since, as we mentioned above, our main analyses in which we used mixed-effect logistic regressions required a dependent variable per trial, we could not use these measurements for our main analyses.

The measurement of violation of WASRP considers only the Decoy condition (excluding trials in which the *decoy* option was chosen). It compares between the probability of choosing the *target* when the *decoy* was asymmetrically dominated by the *target* and the probability of choosing the *target* when it was asymmetrically dominated by the *competitor*. Thus, a demonstration of the decoy effect for each option (A/B) is when:

1. *The probability of choosing A is greater in {A, B, A'} than in {A, B, B'}*
2. *The probability of choosing B is greater in {A, B, B'} than in {A, B, A'}.*

Where *A′* represents a *decoy,* which is asymmetrically dominated by option A, and *B′* represents a *decoy* which is asymmetrically dominated by option B.

The measurement of RST considers only the Decoy condition as well, however it takes into account also trials in which the *decoy* option was chosen. This is the calculation of RST per subject:

$$\frac{\Pr(T)}{\Pr(T) + \Pr(C)}$$

Where Pr(T) is the choice proportion of the *target*, and Pr(C) is the choice proportion of the *competitor*.

The measurement of violation of regularity compares between the probability of choosing the *target* in the Decoy condition (trinary choice, excluding trials in which the *decoy* option was chosen) and the probability of choosing the *target* in the Basic condition (binary choice). A demonstration of the decoy effect for each option (A/B) is when:

1. *The probability of choosing A is greater in {A, B, A'} than in {A, B}.*
2. *The probability of choosing B is greater in {A, B, B'} than in {A, B}.*

Where *A′* represents a *decoy* which is asymmetrically dominated by option A, and *B′* represents a *decoy* which is asymmetrically dominated by option B.

It is important to note that both WASRP violation and RST allow a combination of both options A and B as a *target*, while the regularity violation do not. This is because there is no definition of a *target* option in the Basic condition. Therefore, we compared our measurement (choice proportion of *target*) only to WASRP violation and RST. Nonetheless, we present here the measurement of the *attraction effect* using the regularity violation with our data, as well.

Experiment 1

As was mentioned above, violation of WASRP compares between the probability of choosing the *target* when the *decoy* was asymmetrically dominated by the *target* and the

probability of choosing the *target* when it was asymmetrically dominated by the *competitor*. We calculated the WASRP violation separately for each option.

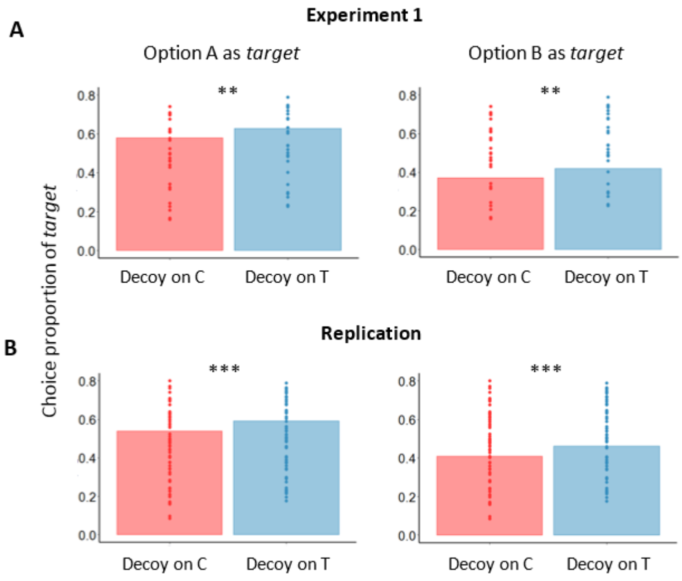

Fig 1. WASRP violation results. The probability of choosing the *target* was significantly higher when the *decoy* was asymmetrically dominated by the *target* (blue bars) than when it was asymmetrically dominated by the *competitor* (pink bars). The dots in each bar represent individual subjects. **(A) Experiment 1:** mean difference=0.047, CI=0.013-0.082, p<0.01. **(B) Replication:** mean difference=0.053, CI=0.027-0.079, p<0.001.

As can be seen Fig 1A, subjects displayed a significant violation of WASRP for both options. That is, the probability of choosing the *target* was significantly higher when the *decoy* was asymmetrically dominated by the *target* than when it was asymmetrically dominated by the *competitor* (Both option A and option B: mean difference=0.047, CI=0.013-0.082, p<0.01).

Similarly to our main dependent measurement (choice proportion of *target*), there was a considerable heterogeneity between subjects in WASRP violation. Only 75% of the subjects displayed a positive difference which means that they demonstrated an *attraction effect*, while the remaining 25% of the subjects displayed the opposite effect (a *repulsion effect*). The range of differences spreads between -0.25 and 0.35 (Fig. 2A).

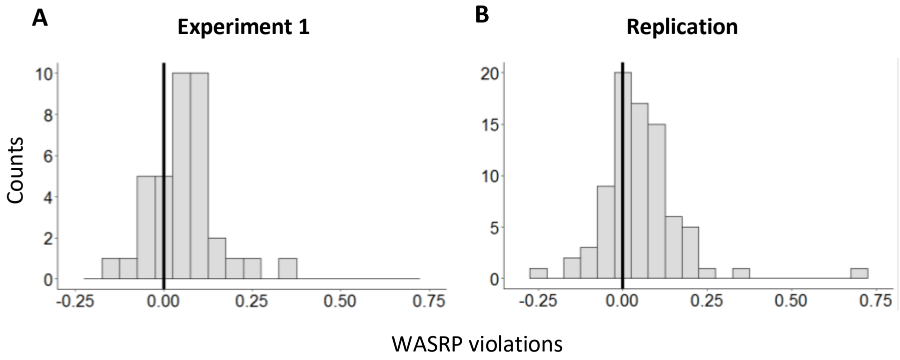

Fig 2. Histogram of WASRP violations: the mean differences in the probability of choosing the *target* when the decoy was asymmetrically dominated by the *target* and when it was asymmetrically dominated by the *competitor*. The black line represents a WASRP violation of zero. The negative WASRP violations (on the left side of the zero line) represent subjects who displayed a general *repulsion effect,* while the positive WASRP violations (on the right side of the zero line) represent subjects who displayed a general *attraction effect.* **(A) Experiment 1:** mean difference=0.047, n = 38. **(B) Replication:** mean difference=0.053, n = 81.

Moreover, there is a very high positive correlation between the choice proportion of *target* and the WASRP violation (R=0.99, p<0.001; Fig. 3A), indicating that our main dependent measurement (the probability to choose the *target* in each trial) is similar to the measurement of WASRP violation.

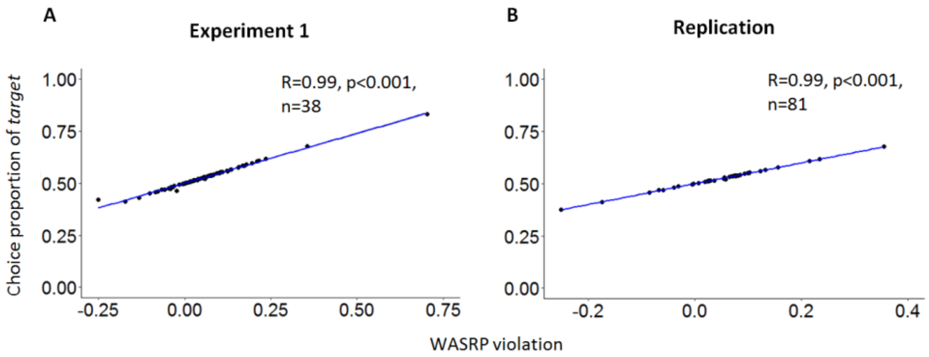

Fig 3. Correlation between WASRP violation and choice proportion of *target*. There is a significantly positive correlation between the two measurements of the *attraction effect* size: WASRP violation and the probability to choose the *target*. **(A) Experiment 1:** R=0.99, p<0.001, n=38. **(B) Replication:** R=0.99, p<0.001, n=81.

Furthermore, there was a very high positive correlation between the choice proportion of *target* and the RST measurement (R=0.97, p<0.001; Fig. 4A), indicating that our main

dependent measurement (choice proportion of *target*) is similar to the RST measurement as well, and that excluding the trials in which the *decoy* option was chosen did not influence our results in the main analyses.

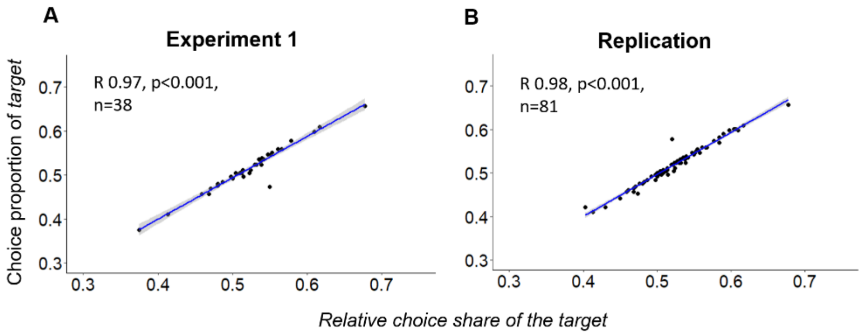

Fig 4. Correlation between RST and choice proportion of *target*. There is a significantly positive correlation between the two measurements of the *attraction effect* size: RST and choice proportion of *target*. **(A) Experiment 1:** R=0.97, p<0.001, n=38. **(B) Replication:** R=0.98, p<0.001, n=81.

Regarding regularity violation, subjects showed higher probability to choose the *target* both for option A and option B in the Decoy condition in comparison with the Basic condition. However, this increase did not reach significance for both options (**option A:** mean difference=0.02, CI=-0.03-0.07, p=0.21; **option B:** mean difference=0.03, CI=-0.02-0.07, p=0.21; Fig. 5A). These results are in line with previous studies which have shown that regularity violation appears to be weaker than WASRP violation [8, 2]. A possible reason for this is that WASRP is a stronger requirement and necessarily implies the regularity condition [8].

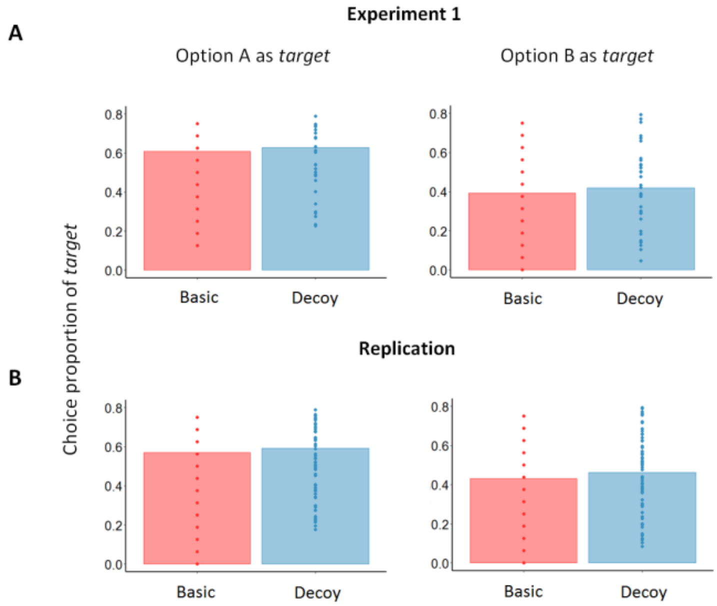

Fig 5. Regularity violation results. Subjects showed higher probability to choose the *target* both for option A and option B in the Decoy condition in comparison with the Basic condition. However, this increase did not reach significance in both experiments. **(A) Experiment 1: option A:** mean difference=0.02, CI=-0.03-0.07, p=0.21; **option B:** mean difference=0.03, CI=-0.02-0.07, p=0.42. **(B) Replication: option A:** mean difference=0.02, CI=-0.008-0.05, p=0.16; **option B:** mean difference=0.032, CI=-0.007-0.07, p=0.08.

Replication

Similar to Experiment 1, the subjects displayed a significant violation of WASRP for both options (mean difference=0.053, CI=0.027-0.079, p<0.001; Fig. 1B). Interestingly, in both experiments, the mean difference in the probability of choosing the *target* between the two conditions was ~0.05 (Fig. 1). Moreover, as in Experiment 1, we observed a considerable heterogeneity between subjects in their difference in the probability of choosing the *target* between the two conditions (Fig. 2B), and there was the same proportion of subjects (25%) that displayed a *repulsion* effect with a similar spread across subjects (-0.25 and 0.75). Lastly, similar to Experiment 1, we found a high positive correlation between the choice proportion of the *target* and the WASRP violation (R=0.99, p<0.001, n=81; Fig. 3B), indicating that both of these measurements of the *attraction effect* are similar to each other.

Moreover, similar to Experiment 1, there was a high positive correlation between the choice proportion of the *target* and the RST (R=0.98, p<0.001, n=81; Fig. 4B), indicating that both of these measurements of the *attraction effect* are similar to each other.

Regarding the violation of regularity measurement, subjects showed a higher probability to choose the *target* both for option A and option B in the Decoy condition in comparison with the Basic condition. However, as in Experiment 1, this increase did not reach significance for both options (**option A:** mean difference=0.02, CI=-0.008-0.05, p=0.16**; option B:** mean difference=0.032, CI=-0.007-0.07, p=0.08; Fig. 5B).

2. Confound analyses

We performed further analyses in order to exclude the possibility that the significant negative link between the Gestalt threshold and the probability to choose the *target* is merely due to task engagement, such that subjects who were less engaged in the Gestalt task (and thus have higher thresholds) were also less engaged in the Decoy task (and thus have lower *attraction effect* sizes). In the perceptual task, in order to examine task engagement, we measured the slope of the logistic regression fit for each subject. The meaning of the slope of the logistic fit is how accurate was the subject in general, across all intervals (distribution of error rates across trial difficulties). That is, a flat curve means a fully random subject, while the steeper the slope, the more accurate the subject is, with a step function being perfectly accurate. Importantly, in our main analyses (Tables 2-4 in the main text; Fig. 3 and 6 in the main text), as a first step, we excluded subjects with a slope that was not significantly ($p<0.05$) higher than 0, meaning that subjects with a uniform distribution of errors across the different difficulties (very low slope) were excluded from the analyses because they were not engaged in the task (Experiment 1: 4 subjects were excluded, Replication: 8 subjects were excluded).

Moreover, when we used the Gestalt slope as a predictor in our main analysis instead of the Gestalt threshold, we had no significant effect of the Gestalt slope on the probability to choose the *target* in both experiments (Experiment 1: $\beta=0.12$, $p=0.52$; Replication: $\beta=0.04$, $p=0.67$; Table 1). These results indicate that there is no systematic effect of the error rates (task engagement) in the Gestalt task and the level of choice proportion of the *target* in the Decoy task. Hence, this strengthens the notion that task engagement was not the driver of the main relationship we found between the two tasks.

Table 1. Summary of the mixed effects logistic regression model for variables predicting the choice proportion of the *target*.

	Experiment 1 (n = 38)				Replication (n = 81)			
**Fixed-effect Parameters**	***B***	**SE#**	***Z***	**p-val**	***B***	**SE#**	***Z***	**p-val**
Constant	0.15	0.07	1.93	.05	0.15	0.05	3.12	<.01**
Value distance	-0.25	0.14	-1.83	.06	-0.17	0.09	-1.80	.07
Gestalt Slope	0.12	0.17	0.64	.52	0.04	0.09	-2.72	.67
**Random-effects Parameters**	**var**				**var**			
Constant	0.00				0.00			
Value distance	0.14				0.16			

Robust Std. Err. (Errors clustered by Subject); * p<.05 **p<.01 *** p<.001

Regarding the Decoy task, it is impossible to define a choice error since there is no correct answer in each trial (except for the first order stochastically dominated trials in which all the subjects, except one, chose the 100% winning probability options all the time). Nonetheless, equivalently to the slopes of the logistic fits in the Gestalt task, we measured the choice variance in each trial type in the Decoy task. There were 32 different trial types (2 different data sets, 2 attributes (probability/amount), 2 different types of decoy (range/frequency), 4 options of VD), which were repeated 8 times each. On the one hand, we expect that a subject who is engaged in the task, will have a relatively small variance in her choices across repetitions of the exact same trial. On the other hand, analogous to the Gestalt task, we would also expect that a subject who is engaged in the task will have some variance across the different trial types (different levels of *decoy*).

Therefore, we calculated two measurements: 1) the mean of choice variance, where we calculated the variance in choice of the 8 repetitions for each trial type, and then averaged for each subject these variances across the 32 trial types (*equation 1*). This average of choice variance gives an indication of how consistent was the subject per trial type (the smaller this average of choice variance, the more consistent was the subject per trial type and thus, more engaged in the Decoy task), and 2) the variability across trial types (variance of means), where we calculated the probability to choose a specific option (A/B) per trial type and then calculated

the variability of choice probabilities across trial types (*equation 2*). This measurement represents if the subject responded differently across the different trial types (the smaller the variability of choices across trial types, the less the subject changed his response according to the different trial types, and thus, we assume, the less engaged she was in the task).

Equation 0:

$$\frac{\sum_{1}^{Ni} x_{ijk}}{N_i} = P_{jk}$$

P_{jk} is the choice probability of option B^* across the 8 repetitions of a trial type, where *i* stands for repetition (8 per trial type), *j* stands for trial type (32 per subject) and *k* stands for subject.

Equation 1:

$$\frac{\sum_{1}^{Nj} P_{jk}(1 - P_{jk})}{N_j} = X_k$$

X_k is the average of choice variance. We first calculated the variance across the repetitions per each trial type, and then calculated the average of these variances for each subject.

Equation 2:

$$\frac{\sum_{1}^{Nj}(P_{jk} - \overline{P_k})^2}{N_j - 1} = Y_k$$

Y_k is the variability across trial types (variance of means). We first calculated the choice probability across the repetitions per each trial type, and then calculated the variance of these choice probabilities for each subject. $\overline{P_k}$ stands for the mean of choice proportion of option B per subject.

* We randomly chose option B. It is equivalent for both options (A and B).

If both of these measurements are indications for task engagement, we would assume a negative connection between them: the smaller the mean of choice variance per trial type, the higher the variability of the choices across trial types (which would indicate on a more engaged subject). Interestingly, this is exactly what we observed when we correlated between these

measurements (Fig. 6): the X axis represents the mean of choice variance per trial type (X_k in *equation 1*), and the Y axis represents the variability of choices across trial types (Y_k in *equation 2*) [Experiment 1: R=-0.46, p<0.01, n=38; Replication: R=-0.63, p<0.001, n=81].

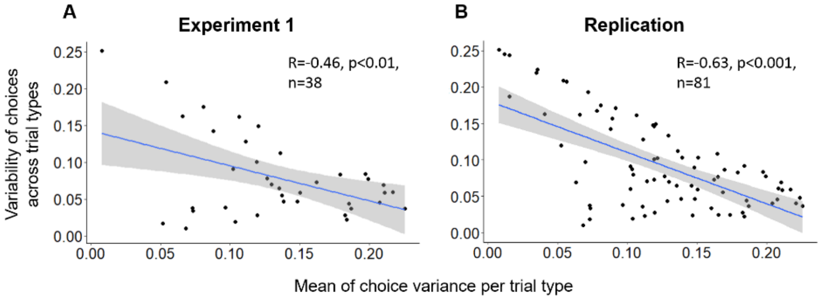

Fig 6. Correlation between the two measurements for task engagement in the Decoy task: the mean of choice variance per trial type and the variability of choices across trial types. There is a significant negative correlation between the mean of choice variance per trial type and the variability of choices across trial types in both experiments. **(A) Experiment 1:** R=-0.46, p<0.01, n=38. **(B) Replication:** R=-0.63, p<0.001, n=81.

In order to examine a potential connection between task engagement in the Decoy task and the tendency to show an *attraction effect*, we examined the correlation between each of these measurements for task engagement (the mean of choice variance per trial type and the variability of choices across trial types) and the choice proportion of the *target*.

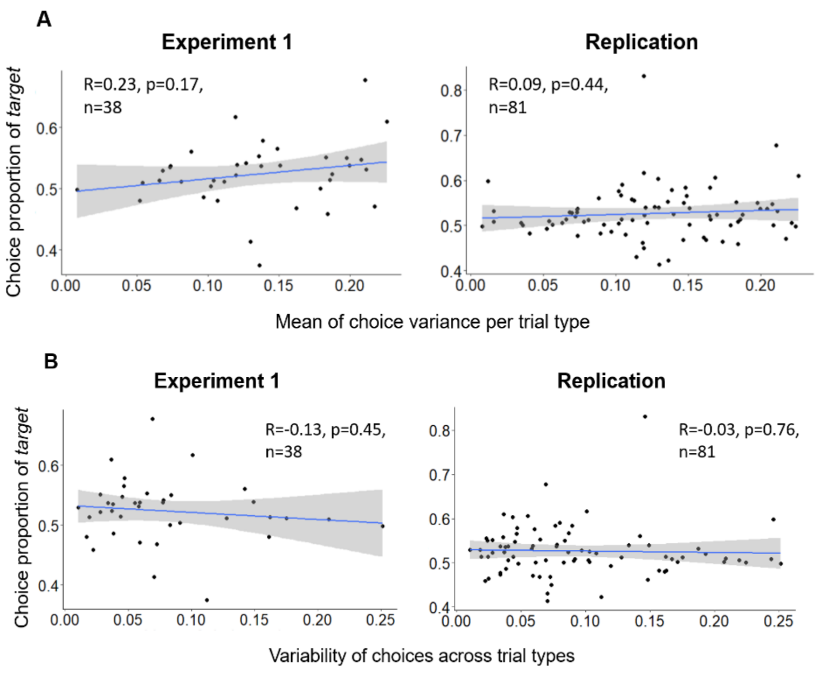

Fig 7. Correlation between each of the two measurements for task engagement in the Decoy task and choice proportion of *target*. (A) There is no significant correlation between the mean of choice variance per trial type and the choice proportion of the *target* in both experiments (Experiment 1: R=0.23, p=0.17, n=38; Replication: R=0.09, p=0.44, n=81). **(B)** There is no significant correlation between the variability of choices across trial types and the choice proportion of the *target* in both experiments (Experiment 1: R=-0.13, p=0.45, n=38; Replication: R=-0.03, p=0.76, n=81).

As shown in Fig. 7, there is no significant correlation between the mean of choice variance per trial type and the choice proportion of the *target* in both experiments (Experiment 1: R=0.23, p=0.17, n=38; Replication: R=0.09, p=0.44, n=81; Fig. 7A). Additionally, there is no significant correlation between the variability of choices across trial types and the choice proportion of the *target* in both experiments (Experiment 1: R=-0.13, p=0.45, n=38; Replication: R=-0.03, p=0.76, n=81; Fig. 7B). These results indicate that subjects who had a higher variance in their choices per trial type or a small variability across trial types, and thus were probably less engaged in the Decoy task, did not choose systematically the *target* more or less often.

Moreover, in order to test if there is a connection between the level of task engagement in both tasks, we examined the correlation between each of the two measurements for task engagement in the Decoy task (the mean of choice variance per trial type and the variability

across trial types) and the measurement of task engagement for the Gestalt task (the slope of the logistic fit). We found no significant correlation between the mean of choice variance per trial type in the Decoy task and the Gestalt slope in both experiments (Experiment 1: R=0.16, p=0.32, n=38; Replication: R=-0.12, p=0.27, n=81; Fig. 8A) as well as no significant correlation between the variability across trial types in the Decoy task and the Gestalt slope (Experiment 1: R=-0.18, p=0.28, n=38; Replication: R=0.09, p=0.42, n=81; Fig. 8B), which demonstrates that subjects who were less engaged in the Decoy task were not necessarily less engaged in the Gestalt task as well. Hence, this strengthens our interpretation regarding the relation that we found between the two tasks and indicates that it is not caused by the lack of task engagement.

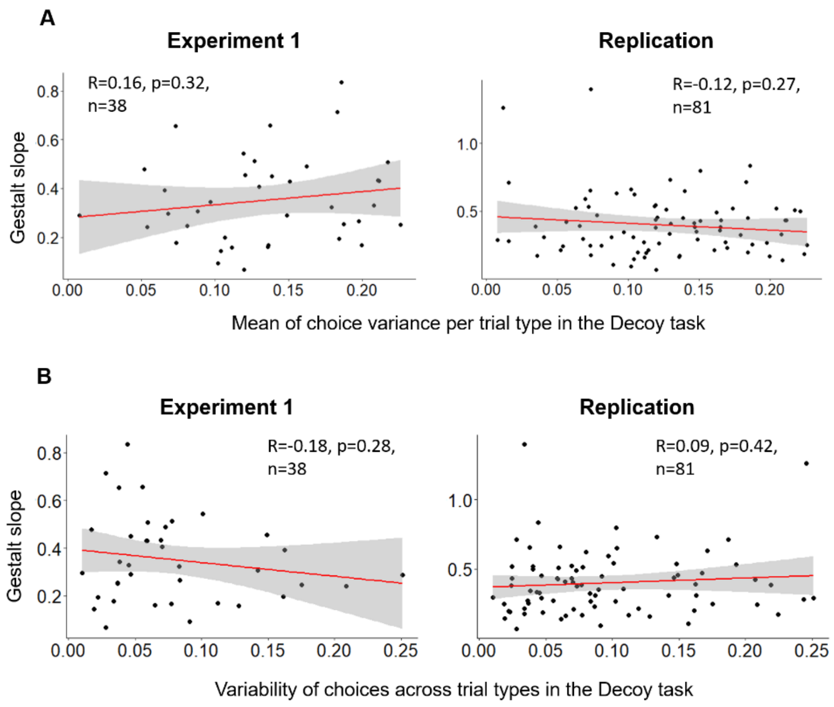

Fig 8. Correlation between each of the two measurements for task engagement in the Decoy task and the measurement for task engagement in the Gestalt task (the slope of the logistic fit). (A) There is no significant correlation between the mean of choice variance per trial type in the Decoy task and the slope of the logistic fit in the Gestalt task (Experiment 1: R=0.16, p=0.32, n=38; Replication: R=-0.12, p=0.27, n=81). **(B)** There is no significant correlation between the variability of choices across trial types in the Decoy task and the slope of the logistic fit in the Gestalt task (Experiment 1: R=-0.18, p=0.28, n=38; Replication: R=0.09, p=0.42, n=81).

Finally, we ran a regression analysis which includes VD, Gestalt threshold, and the task engagement measurements (Gestalt slope for the Gestalt task and the mean of choice variance per trial type for the Decoy task) as predictors to the choice proportion of the *target* for both experiments in order to examine if there is any overall influence of task engagement on our main results [we did not include the variability of choices across trial types as a predictor since there is a significant correlation between it and the mean of choice variance per trial type].

Table 2. Summary of the mixed effects logistic regression model for variables predicting the choice proportion of the target including task engagement measurements.

	**Experiment 1 (n = 38)**				**Replication (n = 81)**			
**Fixed-effect Parameters**	**B**	**SE#**	**Z**	**p-val**	**B**	**SE#**	**Z**	**p-val**
Constant	0.40	0.18	2.23	<.05*	0.44	0.12	3.78	<.001***
Value distance	-0.25	0.14	-1.86	.06	-0.17	0.09	-1.82	.07
Gestalt Threshold	-0.04	0.02	-2.06	<.05*	-0.03	0.01	-2.69	<.01**
Mean of choice variance per trial type	0.26	0.54	0.48	.63	-0.22	0.35	-0.64	.52
Gestalt Slope	0.03	0.17	0.15	.88	-0.03	0.09	-0.36	.72
**Random-effects Parameters**	**var**				**var**			
Constant	0.00				0.00			
Value distance	0.14				0.16			

Robust Std. Err. (Errors clustered by Subject); * p<.05 **p<.01 *** p<.001

As presented in Table 2, none of the task engagement measurements had a significant effect on the choice proportion of the *target* in both experiments. Furthermore, the coefficients of our main predictors (Gestalt threshold and VD) of the model which includes the task engagement measurements (Table 2) were very similar to the coefficients of our main predictors in our main model in the paper (Table 4 in the main text) in both experiments. This result suggests that the effect of the Gestalt sensitivity on choice is not related to task engagement.

In sum, these results demonstrate that the significant negative link between the Gestalt thresholds and the choice proportion of *target* is not merely due to the level of engagement in both tasks.

3. Higher choice proportion of the safer option

It is important to note that although we aimed to reach an indifference between options A and B using the Calibration task, the safer option (option A) was chosen more often across subjects in the Basic condition in both experiments, albeit only significant in the Replication experiment (Experiment 1: mean choice proportion of option A: 0.61, $t(38)=1.89$, $p=0.07$; Replication: mean choice proportion of option A: 0.57, $t(81)=2.85$, $p<0.01$; Fig. 5 and Fig. 9). There is a very wide range of preferences of option A in both experiments (Experiment 1: from 0.125 to 1, Replication: from 0 to 1; Fig 9). Additionally, we conducted a binomial test to examine the significance of the preference towards a specific option per subject. In both experiments, ~30% of the subjects significantly preferred one of the options (Experiment 1: 26% of the subjects significantly preferred option A over B and none of the subjects significantly preferred option B; Replication: 32% of the subjects significantly preferred one of the options, and 70% out of these subjects significantly preferred option A over B). This indicates that although we aimed for subjects to be indifferent in the subjective value between option A and B by using the Calibration task, around third of the subjects had a significant difference in the subjective value between the options. A previous study demonstrated that an increase in the subjective value difference between the relevant options (A and B) leads to a decrease in the *attraction effect* [10]. Therefore, this could be one of the reasons for the small *attraction effect* sizes in our study. Nonetheless, although the calibration task did not work perfectly, we were able to show a significant *attraction effect* across subjects as well as a significant link between the choice proportion of the *target* and the susceptibility to group by proximity.

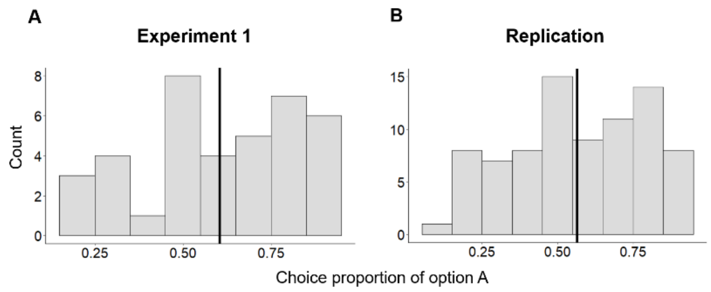

Fig 9. Histogram of the probability to choose the safer option (option A) in the Basic condition: The black line represents the mean of the choice proportion of option A across subjects. **(A) Experiment 1:** mean = 0.61, n = 38. **(B) Replication:** mean = 0.57, n = 81.

4. Model comparisons

To further examine the robustness of our regression analyses, we also performed likelihood ratio tests (LRT) to compare between models.

First, we compared between a mixed-effects logistic regression which includes only an intercept (M0) and a mixed-effects logistic regression which includes the value distance (VD) as a predictor as well (M1). As shown in Table 3, the addition of the VD as a predictor to the regression model produced a marginal significant effect in both experiments (Experiment 1: Chisq=3.29, p=0.07; Replication: Chisq=3.22, p=0.07). Moreover, the AIC score of M1 was (slightly) better than the AIC score of M0. This indicates that the addition of VD to the model increased (slightly) the goodness-of-fit.

$$\text{M0: } \mathrm{P(Choice\ Target)_{ij}} = \frac{1}{1+\exp^{(-(\beta 0j + \varepsilon_{ij})}}$$

$$\text{M1: } \mathrm{P(Choice\ Target)_{ij}} = \frac{1}{1+\exp^{(-(\beta 0j + \beta 1j\,\textbf{Value distance} + \varepsilon_{ij})}}$$

Table 3. Summary of model comparisons to examine the significance of adding Value distance.

	Experiment 1 (n = 38)						Replication (n = 81)					
models	AIC	logLik	deviance	Chisq	Chi Df	Pr(>Chisq)	AIC	logLik	deviance	Chisq	Chi Df	Pr(>Chisq)
M0	13260.74	6626.371	13252.74				28287.47	14139.74	28279.47			
M1	13259.45	6624.723	13249.45	3.295668	1	0.06946285	28286.25	14138.13	28276.25	3.21966	1	0.07275873

Moreover, we compared between a mixed-effects logistic regression which includes only VD as a predictor of the probability to choose the target (M1) and a mixed-effects logistic regression which includes both VD and Gestalt threshold as predictors (M2). As shown in Table 4, the addition of the Gestalt threshold to the regression model produced a significant effect in both experiments (Experiment 1: Chisq=5.00, p<0.05; Replication: Chisq=7.21, p<0.01), and the AIC score was lower in M2 compared to M1.

$$\text{M1: } \mathrm{P(Choice\ Target)_{ij}} = \frac{1}{1+\exp^{(-(\beta 0j+\beta 1j\mathbf{Value\ distance}\ +\varepsilon_{ij})}}$$

$$\text{M2: } \mathrm{P(Choice\ Target)_{ij}} = \frac{1}{1+\exp^{(-(\beta 0j+\beta 1j\mathbf{Value\ distance}\ +\ \beta 2\ \mathbf{Gestalt\ threshold}+\varepsilon_{ij})}}$$

Table 4. Summary of model comparisons to examine the significance of adding Gestalt threshold.

	Experiment 1 (n = 38)						Replication (n = 81)					
**models**	**AIC**	**logLik**	**deviance**	**Chisq**	**Chi Df**	**Pr(>Chisq)**	**AIC**	**logLik**	**deviance**	**Chisq**	**Chi Df**	**Pr(>Chisq)**
**M1**	13259.45	-6624.723	13249.45				28286.25	-14138.13	28276.25			
**M2**	13256.44	-6622.222	13244.44	5.002777	1	0.02530668	28281.04	-14134.52	28269.04	7.212329	1	0.00724045

Furthermore, we compared between a mixed-effects logistic regression which includes only Gestalt threshold as a predictor of the probability to choose the target (M3) and a mixed-effects logistic regression which includes both VD and Gestalt threshold as predictors (M2). As shown in Table 5, the addition of the VD to the regression model that already includes the Gestalt threshold produced only a marginal significant effect in both experiments (Experiment 1: Chisq=3.43, $p<0.05$; Replication: Chisq=3.32, $p<0.01$).

$$\text{M3: } \mathrm{P(Choice\ Target)_{ij}} = \frac{1}{1+\exp^{(-(\beta 0j+\beta 1j\mathbf{Gestalt\ threshold}\ +\varepsilon_{ij})}}$$

$$\text{M2: } \mathrm{P(Choice\ Target)_{ij}} = \frac{1}{1+\exp^{(-(\beta 0j+\beta 1j\mathbf{Value\ distance}\ +\ \beta 2\ \mathbf{Gestalt\ threshold}+\varepsilon_{ij})}}$$

Table 5. Summary of model comparisons to examine the significance of adding Value distance as an addition to the Gestalt threshold.

	Experiment 1 (n = 38)						Replication (n = 81)					
**models**	**AIC**	**logLik**	**deviance**	**Chisq**	**Chi Df**	**Pr(>Chisq)**	**AIC**	**logLik**	**deviance**	**Chisq**	**Chi Df**	**Pr(>Chisq)**
**M3**	13257.87	-6623.935	13247.87				28282.36	-14136.18	28272.36			
**M2**	13256.44	-6622.222	13244.44	3.427645	1	0.06411347	28281.04	-14134.52	28269.04	3.31989	1	0.06844643

These results demonstrate that using model comparisons (LRT) yielded similar results as using the significance of slopes (Wald test). The VD has a marginal significant effect when added to either a null model which includes only an intercept as a predictor or a model which

includes only the Gestalt threshold as a predictor. Additionally, the Gestalt threshold has a significant effect when added to a model that includes only the VD as a predictor. These results strengthen our main conclusion that there is a connection between the sensitivity to the proximity law (Gestalt threshold) and the *attraction effect* (choice proportion of *target*).

Supplementary References

1. Bandyopadhyay T, Dasgupta I, Pattanaik PK. Stochastic revealed preference and the theory of demand. Journal of Economic Theory. 1999;110: 95–110.

2. Castillo G. The attraction effect and its explanations. Games and Economic Behavior. 2020;119: 123-147.

3. Tversky A. Prospect theory: an analysis of decision under risk. Psychological Review. 1972;79(4): 281-99.

4. Spektor MS, Kellen D, Hotaling JM. When the good looks bad : an experimental exploration of the repulsion effect. Psychological Science. 2018;29(8): 1309-20.

5. Berkowitsch NAJ, Scheibehenne B, Rieskamp J. Rigorously testing multialternative decision field theory against random utility models. J Exp Psychol Gen. 2014;143(3):1331–48.

6. Trueblood JS, Brown SD, Heathcote A, Busemeyer JR. Not just for consumers : context effects are fundamental to decision making. Psychological Science. 2013;24(6): 901-908.

7. Mohr PNC, Heekeren HR, Rieskamp J. Attraction effect in risky choice can be explained by subjective distance between choice alternatives. Sci Rep. 2017;(7): 1–10.

8. Dasgupta I, Pattanaik PK. 'Regular' choice and the weak axiom of stochastic revealed preference. Economic Theory. 2007;31: 35–50.

9. Liew SX, Howe PDL, Little DR. The appropriacy of averaging in the study of context effects. Psychon Bull Rev. 2016;1: 1639–46.

10. Farmer GD, Warren PA, El-Deredy W, Howes A. The Effect of Expected Value on Attraction Effect Preference Reversals. J Behav Decis Mak. 2017;30(4):785–93.
